# The Development of Context-Sensitive Attention Across Cultures: The Impact of Stimulus Familiarity

**DOI:** 10.3389/fpsyg.2020.01526

**Published:** 2020-07-10

**Authors:** Solveig Jurkat, Moritz Köster, Relindis Yovsi, Joscha Kärtner

**Affiliations:** ^1^ Department of Psychology, University of Münster, Münster, Germany; ^2^ Institute of Psychology, Free University Berlin, Berlin, Germany; ^3^ Independent Consultant, Brussels, Belgium

**Keywords:** visual scene perception, holistic and analytic perception, eye-tracking, stimulus familiarity, cross-cultural research

## Abstract

Across cultures, there are marked differences in visual attention that gradually develop between 4 and 6 years of age. According to the social orientation hypothesis, people in interdependent cultures should show more pronounced context sensitivity than people in independent cultures. However, according to the differential familiarity hypothesis, the focus on the salient object should also depend on the familiarity of the stimulus; people will focus more on the focal object (i.e., less context sensitivity), if it is a less familiar stimulus. To examine the differences in visual attention between interdependent and independent cultures while taking into account stimulus familiarity, this study used an eye-tracking paradigm to assess visual attention of participants between 4 and 20 years who came from urban middle-class families from Germany (*n* = 53; independent culture) or from Nso families in a rural area in Cameroon (*n* = 50; interdependent culture). Each participant saw four sets of stimuli, which varied in terms of their familiarity: (1) standard stimuli, (2) non-semantic stimuli, both more familiar to participants from Germany, (3) culture-specific matched stimuli, and (4) simple stimuli, similarly familiar to the individuals of both cultures. Overall, the findings show that mean differences in visual attention between cultures were highly contingent on the stimuli sets: In support of the social orientation hypothesis, German participants showed a higher object focus for the culture-specific matched stimuli, while there were no cultural differences for the simple set. In support of the differential familiarity hypothesis, the Cameroonian participants showed a higher object focus for the less familiar sets, namely the standard and non-semantic sets. Furthermore, context sensitivity correlated across all the sets. In sum, these findings suggest that the familiarity of a stimulus strongly affects individuals’ visual attention, meaning that stimulus familiarity needs to be considered when investigating culture-specific differences in attentional styles.

## Introduction

The way in which people attend to their visual field differs strongly between cultures. This has been demonstrated by various cross-cultural studies that have mainly focused on educated urban middle-class participants in Western and Eastern cultural contexts (e.g., Masuda and Nisbett, 2001; Nisbett et al., 2001; Nisbett and Masuda, 2003). In particular, Masuda and Nisbett (2001) describe two prototypical attentional styles: a holistic style with a higher context sensitivity and the focus on the broader perceptual field; and an analytic style in which the focus is on salient objects and their characteristics. While the holistic style has been described as being typical for East Asian adults, the analytic style is considered to be more typical for Western adults. In order to explain where these differences originate, it has been proposed that these cultural differences in cognitive patterns are due to differences in the social orientation of the participants (Markus and Kitayama, 1991; Nisbett et al., 2001; Varnum et al., 2010). That is, while independent cultures endorse autonomy and consider the self as separate from others, interdependent cultures emphasize relatedness and interconnection of the self and others (Triandis, 1989; Markus and Kitayama, 1991). These differences in social orientation are considered to be a driving force behind the differences in cognitive patterns, which explains why interdependent cultural contexts, such as East Asian societies, show a more holistic style, and independent cultural contexts, such as Western societies, show a more analytic style (Masuda et al., 2019; Nisbett et al., 2001). There are also studies outside the East-West dichotomy that support the assumption that cultures differing in social orientation also show corresponding differences in cognitive styles (e.g., Kitayama et al., 2006; Knight and Nisbett, 2007; Uskul et al., 2008). For example, Uskul et al. (2008) compared different types of subsistence, namely farmers, fishers, and herders within Turkey. The authors could demonstrate that communities that are characterized by group collaboration that fosters greater interdependence, such as farming and fishing communities, show a more holistic cognitive style than communities that tend to emphasize individual decision-making and social independence, such as herding communities (Uskul et al., 2008).

Cultural differences in attentional styles have been reported consistently across a variety of tasks, such as picture description and recognition tasks (Masuda and Nisbett, 2001), change blindness (Masuda and Nisbett, 2006), and eye-tracking paradigms (e.g., Chua et al., 2005). For example, Chua et al. (2005) presented naturalistic pictures with a clear focal object and a background (e.g., tiger in the woods) and found that Chinese graduate students showed higher context sensitivity as they spent more time looking at the background than US-American graduate students.

Empirical evidence indicates that cross-cultural differences in attentional styles start to develop in the late preschool years, around 4 to 6 years, and become further pronounced in the years thereafter (Duffy et al., 2009; Imada et al., 2013). For example, Imada et al. (2013) compared the visual attention of 4- to 9-year-old children from Minneapolis, USA, and Kyoto, Japan, in an optical illusion and picture description task. They showed that children from around 6 to 7 years, but not younger, display culture-specific attentional styles. This means children from Kyoto, Japan, show a more holistic style, as they showed greater illusion and described more often the background, than children from Minneapolis, USA. These differences further increase at 8 to 9 years of age (Imada et al., 2013).

A recent study extended the research on the development of culture-specific attentional styles to cultures other than educated urban middle-class families from the USA or East Asia. Köster et al. (2018) compared the holistic and analytic attention to visual scenes of 5-year-old children from the Nso culture (rural Cameroon), Münster (urban Germany), and Kyoto (urban Japan) in three different tasks, namely eye-tracking, picture description, and an optical illusion. One of their main findings was that the context sensitivity across the different tasks, which were unrelated, was less consistent than suggested by previous studies. Furthermore, while some tasks revealed the expected cultural differences, others pointed in the opposite direction. Looking at the eye-tracking task in more detail, Köster et al. (2018) used two different sets of stimuli to measure children’s spontaneous gaze behavior, namely a set of natural pictures and a set of non-semantic pictures, in which geometric objects were displayed in front of abstract backgrounds. The set of non-semantic stimuli were chosen with the rationale that the stimuli were unfamiliar to children in all three cultures. However, against expectations, the children from rural Cameroon showed a higher object focus for both natural and non-semantic objects than children from urban middle-class families in Japan and Germany. The authors proposed that this reversed pattern may be due to an unfamiliarity effect, because both the natural and non-semantic stimulus types are less common in the Nso children’s lifeworld. More specifically, the animals and vehicles presented in the natural stimuli set do not occur in Nso children’s everyday life and – due to the fact that these children are much less familiar with books or electronic media – also the non-semantic stimuli that roughly resembled comics in everyday life – seemed unfamiliar. This unfamiliarity may have led to an increased interest in the presented objects (Caparos et al., 2012; Bremner et al., 2016).

Familiarity has been considered as an important aspect in memory and cognitive processing (Snodgrass and Vanderwart, 1980), as familiarity of visual scenes facilitates encoding and categorization (Pashler, 1988). Because the physical environment differs profoundly between different cultural contexts (Miyamoto et al., 2006), people are exposed to very different visual information. To date, it has not been systematically tested whether and how the familiarity of stimuli influences participants’ attentional styles in experimental settings and thereby may affect the findings of cross-cultural studies.

The present study set out to close this empirical gap by examining how different types of stimuli, varying in familiarity, affect cross-cultural differences in context sensitivity in an eye-tracking task. Specifically, we were interested in, first, whether perceptual styles are consistent across stimulus categories and, second, whether the familiarity of the stimuli influences the cross-cultural differences in visual attention.

To analyze how the familiarity of stimuli affects cross-cultural differences in analytic and holistic attention, we selected two cultural communities, in particular a middle-class sample from Münster, urban Germany, and a sample from the Nso culture, living in a mainly subsistence-based farming ecology in Kumbo, rural Cameroon. The main reasons for selecting these samples were three-fold: (1) the samples differ in social orientations as described above: they represent an independent and interdependent cultural orientation, respectively; (2) the visual environments in these contexts are highly different; and (3) data exists on the attentional styles for both cultural communities (Köster et al., 2018).

The urban German middle class represents a prototype of an independent (Markus and Kitayama, 1991) – or autonomous (Keller and Kärtner, 2013) – cultural context. Families and household sizes are usually small. Parents are occupied in professional jobs and have high levels of formal education. Parental behavior and socialization focus on dyadic interactions and individual development, such as making choices independently (Kärtner, 2015; Köster et al., 2016). Before children enter school at the age of 6 or 7, they usually attend kindergarten. The majority of children graduate from school at the age of 18 to 19 and then enter University or start to work.

Children from the Nso culture in rural Nsoland in the North West region of Cameroon grow up in large, extended family settings. Livelihood depends on subsistence-based farming which plays a central role for family alimentation. Most parents are farmers and engage their children in household tasks and fieldwork from early on (Köster et al., 2018). This cultural context is considered as prototypically relational (Keller and Kärtner, 2013) and socialization practices focus on taking responsibility associated with social roles in hierarchical social relationships. Child care in this setting becomes a communal responsibility promoting harmonious relationships between family members and the social reference group (Keller, 2007), where from toddlerhood on, children’s life becomes more influenced by peer-groups and siblings than by adults (Nsamenang and Lamb, 1994; Yovsi and Keller, 2003). Nso developmental goals are obedience, conformity, and respect for authority with long-term consequences for developing a cohesive society where members are cooperative, responsible for each other, develop a sense of collective identity and belongingness (Yovsi, 2003). In Kumbo, children attend preschool from 4 to 6 years before they start primary school. School attendance is obligatory and regular from preschool to at least the end of primary school (grade 6 with about 12 years of age). After that, the majority of children attend at least secondary education and only a small minority continues with high school or university.

Given that attentional styles gradually develop during childhood (Imada et al., 2013), the present study covers a wide age range focusing on linear change across age, and captures the early preschool years, when culture-specific attentional styles emerge, to adolescence, when attentional styles should already be fully established. Considering previous findings, we expected that the context sensitivity of participants in both settings would increase with age (Imada et al., 2013).

**Figure 1:**
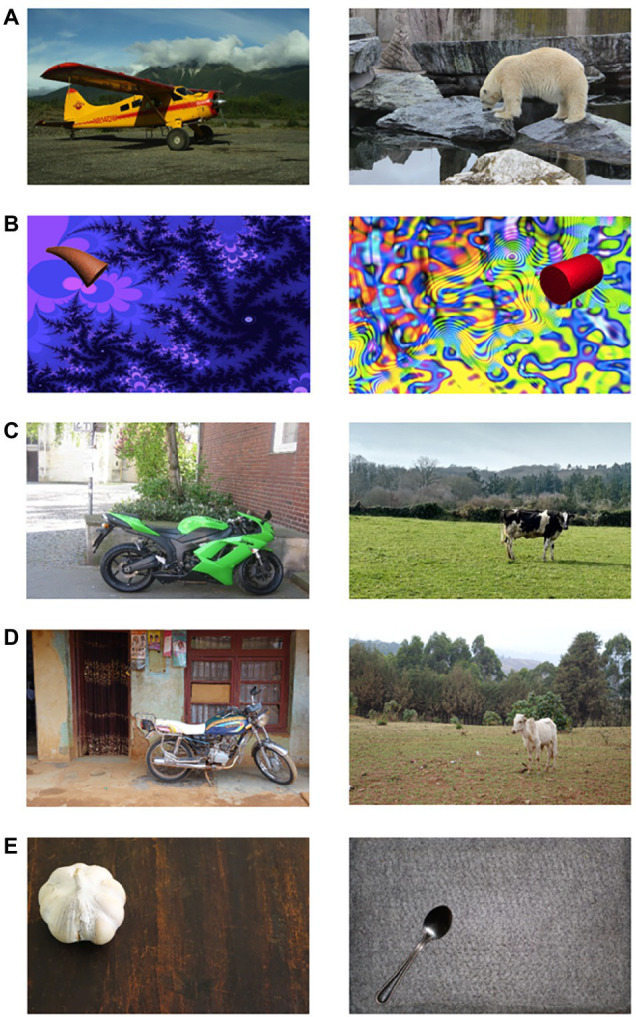
Example stimuli used in the different sets: **(A)** standard set, **(B)** non-semantic set, **(C)** culture-specific matched set in Germany, **(D)** culture-specific matched set in Cameroon, and **(E)** simple set.

Children and adolescents from both cultural contexts participated in an eye-tracking task assessing their spontaneous attention to four different sets of pictures varying in familiarity. The first two sets are similar to the stimuli used in the study by Köster et al. (2018): we used a set of natural pictures and a set of non-semantic stimuli. Both sets should be more familiar to participants in Münster than in Kumbo. As the first set resembles the set that has been used in previous studies with a similar task (e.g., Chua et al. 2005; Köster et al. 2018), we refer to this set as the standard set. In addition, we used a culture-specific matched set, consisting of culturally adjusted, natural stimuli that have been matched across cultures, and we used a set of simple stimuli. These two sets should be equally familiar in Münster and Kumbo (see Figure 1). The standard, culture-specific matched and simple set have been rated by experts from the respective cultural context to back up these assumptions.

Given the social orientation hypothesis – the assumption that in a cultural context, the social orientation is associated with the dominant cognitive style (Varnum et al., 2010) – we hypothesized that rural Nso participants from Kumbo would have a more context sensitive attentional style, spending less time looking at the object, than urban middle-class participants from Münster. Regarding the differential familiarity hypothesis, we expected that this cross-cultural difference in visual attention should be strongest for the sets of stimuli that are equally familiar for both cultures, namely the culture-specific matched set and the simple set. Following the findings from Köster et al. (2018), there might be a less distinct or even reversed pattern for the standard set and the non-semantic set of stimuli.

## Materials And Methods

### Participants

The final sample consisted of 53 participants of middle-class families from Münster in urban Germany and 50 participants of subsistence-based farming families of the Nso-culture from Kumbo in rural Cameroon. The age range was between 4 and 20 years. All participants had normal or corrected to normal visual acuity. In Münster, families were contacted via a database from the local university. In Kumbo, children were recruited by word of mouth. Informed written consent was obtained from parents in both contexts, and children gave informed assent. For their participation, families in Kumbo received financial compensation of 1000 CFA, which was equivalent to 1.50 € at that time. Participants in Münster received cinema vouchers. The type and amount of compensation have been discussed with local assistants to determine a locally appropriate compensation for the time spent.

An additional 12 participants in Kumbo and 15 participants in Münster were not included in the analyses because they did not meet the defined criteria. These were, first, that participants had no more than one degree of deviation when calibrating the eye-tracker and, second, that they were looking at the monitor for at least 70% of the presentation time (indicated by the tracking ratio). Three additional children were excluded because in one case the child did not feel well (Kumbo: *n* = 1) or because of technical problems occurring in the eye-tracking task (Münster: *n* = 2).

The gender distribution did not systematically differ between both samples (64.2% females in Münster, 56.0% females in Kumbo, *χ*^2^ = 1.21, *p* = 0.311). Furthermore, there was no significant age difference between cultures [*M* = 11.75 years, *SD* = 5.6 in Münster, *M* = 10.64 years, *SD* = 4.6 in Kumbo, *t*(101) = −1.099, *p* = 0.274].

### Stimuli and Procedure

Participants attended one experimental session, and each participant saw all four sets of stimuli. In sum, each participant saw 120 pictures: 40 standard, 40 non-semantic, 20 culture-specific matched (either from Münster or Kumbo) and 20 simple pictures. In Kumbo, the laboratory was set up in a quiet room of a house, whereas in Münster, participants visited the university laboratory with a parent. The four stimuli sets differed in familiarity (as described below), and for each set the participant received the instruction to “…watch the pictures as you like…”

#### Standard Set

This set consisted of 40 real pictures displaying a focal object (animals and vehicles) in front of an urban or nature background (see Figure 1A). Pictures were either taken by the authors, obtained from a public domain database^1^ or were selected from the set used by Chua et al. (2005). These pictures are considered to be biased because they are more familiar to participants from Münster than to participants from Kumbo, as some of the depicted objects, such as boats or camels, are well-known to participants from Münster, either from direct experience or from books and other media, while they are much less familiar to participants from Kumbo who have limited access to picture books and other media. Even if animals or vehicles are known, both the object and the background are more familiar to children and adolescents from Münster than to children and adolescents from Kumbo.

#### Non-semantic Set

Further, 40 non-semantic pictures with abstract objects in front of abstract backgrounds were shown (see Figure 1B). We used artificial objects commonly used in experimental psychology (greebles, fribbles, geons, and multipart geons, e.g., Gauthier and Tarr, 1997, taken from an online database: http://wiki.cnbc.cmu.edu/Novel_Objects). Abstract backgrounds were either fractal pictures (Kaspar and König, 2011; created with quadrium 2.0, quadrium.en.softonic.com) or details of an abstract drawing (see Köster et al., 2017). Because these are novel, non-semantic objects and backgrounds, they are unknown in both samples, Münster and Kumbo. However, as suggested by Köster et al. (2018), due to limited access to media, such as books, cartoons, or electronic media, abstract shapes are less familiar to children and adolescents from Kumbo.

#### Culture-Specific Matched Set

These equivalent sets were designed to be similarly familiar in Münster and Kumbo. We compiled two sets of 20 real pictures with animals, vehicles, and buildings as focal objects. One set of pictures was taken in Münster and, therefore, should be highly familiar to children in Münster, and the other set was taken in and around Kumbo. The participants only saw the set with the stimuli from their respective cultural context. Pictures were matched over the two sets in the sense that they depicted the same kind of object in front of an equally complex background (e.g., a typical domestic animal on a typical field, see Figures 1C,D) in the respective setting. Moreover, the position of the salient objects, as well as their size was matched across cultural settings.

#### Simple Set

Finally, we presented a simple set of 20 pictures with common, everyday objects in front of a simple background (e.g., a garlic clove on a wooden table, see Figure 1E), This set was designed to avoid culture-specific compositions in order to retain similar familiarity to children and adolescents from both Münster and Kumbo. The same set of pictures that were taken in and around Kumbo were presented in both contexts, namely to participants in Kumbo and Münster.

#### Familiarity Rating

In order to back up the assumptions concerning the familiarity of the stimuli sets, the pictures of the standard, culture-specific matched and the simple set were rated by experts that were highly familiar with the respective environment. These experts, namely adolescents or adults living in urban Germany (*n* = 5, 100% female, *M_Age_* = 24.20 years) or rural Cameroon (*n* = 6, 50% female, *M_Age_* = 25.00 years) judged the familiarity of the pictures of the standard, culture-specific matched and the simple set for children and adolescents living in their own cultural context. Pictures were presented in randomized order and rated on a 4-point Likert scale (1 = familiar to 4 = unfamiliar). While pictures from these three sets all depict specific objects and backgrounds, which can be easily rated in terms of how common they are in the participants’ everyday life, this was not the case for the abstract shapes in the non-semantic set. Thus, we decided to exclude the non-semantic set from the rating. In support of the assumptions, the standard set was rated as more familiar in Münster (*M* = 2.17, range = 1.30–2.48) than in Kumbo (*M* = 2.88, range = 2.30–3.28), *U* = 3.00, *p* = 0.03. The culture-specific matched pictures were rated as highly familiar in the respective cultural contexts (Kumbo: *M* = 1.01, range = 1.00–1.05; Münster: *M* = 1.25, range = 1.10–1.40), with a value even closer to 1 for the Cameroonian experts, *U* = 0.00, *p* = 0.004. The mean values for the simple set all ranged between familiar and rather familiar for all experts, with higher familiarity ratings by Cameroonian experts (*M* = 1.01, range = 1.00–1.05 vs. Münster: *M* = 2.00, range = 1.55–2.35), *U* = 0.00, *p* = 0.004. Overall, these findings support the assumptions concerning the familiarity of the stimuli sets described above.

Assessments started with either the standard or the culture-specific matched set, with the respective other set being second. Then, participants saw either the non-semantic set or the simple set, with the respective other set being last. The order of the sets was counterbalanced, and pictures were randomized within each set and separated by a blank screen. Following studies that conducted a similar task with children (Köster and Kärtner, 2018; Köster et al., 2018), trials started with a fixation dot (shown for 1 s), followed by the stimulus (5 s). The stimulus presentation procedures were implemented in psychophysics toolbox (Version 3.0.12, on MATLAB, Version R2013a) and with the presentation program ExperimentCenter (Version 3.5.169, SensoMotoric Instruments GmbH, Teltow, Germany). Participants’ gaze behavior was recorded binocularly, at a sampling rate of 250 Hz. Individual fixations were identified using a saccade-based velocity-threshold identification filter with a minimum fixation duration of 50 ms and a saccade peak velocity threshold of 40°/s in the eye-tracking software BeGaze, Version 3.5.101, which is a standard measure used in multiple former studies (e.g., Köster and Kärtner, 2018; Köster et al., 2018). The presentation was on a 15.6-in notebook display in Cameroon and a 24-in desktop monitor in Germany, both with a resolution of 1920 × 1080, but the stimuli were presented with the same dimensions of approximately 20 cm × 32 cm at a distance of 50–70 cm from the participant in both cultural contexts. To calibrate the eye-tracker, participants made saccades to a grid of nine fixation dots on the screen, and four dots were used to validate the calibration results.

Fixations were exported for further analyses in MATLAB (Version 2013a). The BeGaze software was used to define areas of interest (AOIs) around the focal object of each picture to distinguish the focal object from the background. To quantify participants’ visual attention to the object relative to their visual attention to the context, we computed an object score separately for each of the four sets. For each picture, the total duration of all fixations made into the AOI of the object was divided by the duration of all fixations on the picture (object and background area) within the 5 s of stimulus presentation. As a consequence, pictures that were not fixated at all, were not included in the mean score. Based on the object score for each picture, we then calculated the mean object score for each set separately. A score of 1 would mean that the participant only looked at the object, while a score of 0 would indicate that a participant only looked at the background. The lower the mean object score of a participant is, the higher is his or her context sensitivity.

For the culture-specific matched set, where different stimuli were used for the two samples, the average size of the AOIs was slightly larger in the German picture set (*M* = 15.2%, *SD* = 8.2) than in the Cameroonian picture set (*M* = 11.9%, *SD* = 6.1). Since this trend might lead to an increased object focus, we decided to exclude the five pairs of matched pictures in which the differences in object size between the German and the Cameroonian pictures were largest (i.e., between-set difference in the AOIs of at least 9% of the size of the total picture). This resulted in similar average AOI sizes between the German set (*M* = 12.4%, *SD* = 6.9) and the Cameroonian set (*M* = 11.5%, *SD* = 6.5), *t*(28) = −0.365, *p* = 0.718. Thus, the final object score for the culture-specific matched set was based on 15 pictures in each cultural context. For the other sets, the average size of the AOIs was as follows: *M* = 12.8%, *SD* = 7.0 for the standard set; *M* = 6.8%, *SD* = 1.6 for the non-semantic set; and *M* = 13.6%, *SD* = 4.3 for the simple set.

## Results

### Context-Sensitivity Across Sets and Cultures

To test for the cross-cultural and age differences in attentional style and whether these depend on the familiarity of the stimuli used, the object scores, defined as the relative gaze duration on the focal object, for each set were entered as the dependent variable in separate multiple regression analyses. As independent variables, we included culture (1 = Münster, 0 = Kumbo), age (z-standardized) and, to test whether the effect of age was moderated by culture, the interaction term culture × age.

#### Standard Set

**Figure 2:**
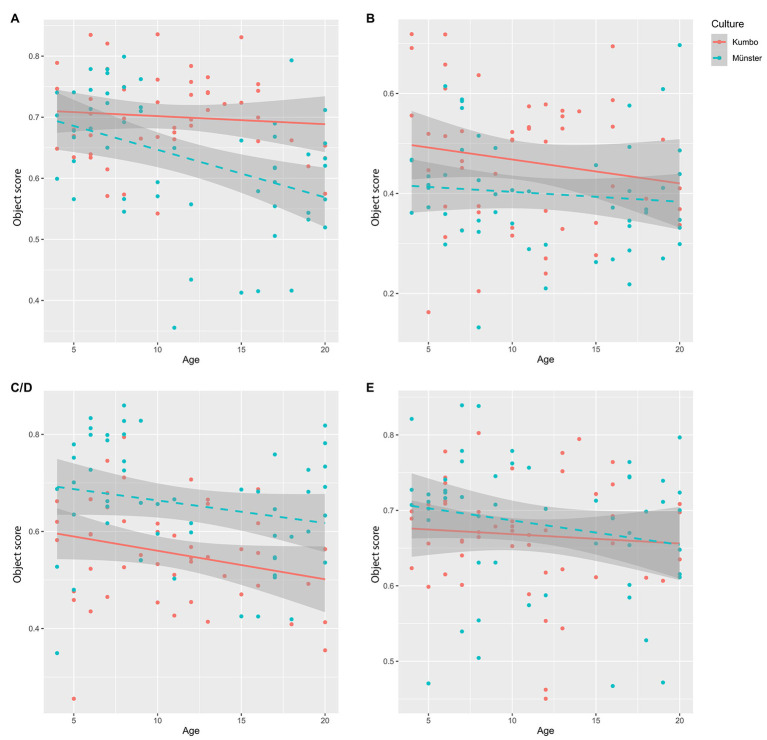
Object scores across culture and age in the (A) standard set, (B) non-semantic set, (C/D) culture-specific matched set, and (E) simple set.

For the standard set, the regression model was significant, *F*(3, 99) = 9.925, *p* < 0.001, with an adjusted *R*^2^ of 0.208. As depicted in Figure 2A, the results indicate that participants from Kumbo had higher object scores than participants in Münster (*β* = −0.327, *p* < 0.001). While there was no significant effect of age (*β* = −0.070, *p* = 0.624), there was a marginally significant age × culture interaction (*β* = −0.270, *p* = 0.061), indicating that developmental changes in context sensitivity differed across cultural contexts. Given the fact that moderator effects are generally difficult to detect, we followed the advice of Pedhazur (1997) and interpret interactions when they achieve the 0.10 level of significance. To determine the simple effects of age, this analysis was followed up by a regression analysis with object score as the dependent and age as the independent variable, separate for the two cultural contexts. While there was a significant regression model in Münster, *F*(1, 51) = 10.277, *p* = 0.002, with an adjusted *R*^2^ of 0.151, in which age was negatively associated with the object scores (*β* = −0.410, *p* = 0.002), the regression model was not significant in Kumbo, *F*(1, 48) = 0.364, *p* = 0.549, *R*^2^*_adj_* = −0.013.

#### Non-semantic Set

For the non-semantic set, there was a significant regression model, *F*(3, 99) = 2.979, *p* = 0.035, with an adjusted *R*^2^ of 0.055. While culture was a significant predictor (*β* = −0.240, *p* = 0.015), neither age (*β* = −0.192, *p* = 0.222) nor the interaction term (*β* = 0.088, *p* = 0.572) were significantly associated with the object score. As depicted in Figure 2B, participants in Kumbo had higher object scores than participants in Münster.

#### Culture-Specific Matched Set

In the culture-specific matched set, we compared the gaze behavior between participants from Kumbo and Münster on their respective culture-specific set. We found a significant regression model, *F*(3, 99) = 8.378, *p* < 0.001, with an adjusted *R*^2^ of 0.178. There was a positive effect of culture on the object score (*β* = 0.421, *p* < 0.001), a marginally significant age effect (*β* = −0.241, *p* = 0.101), but no significant effect of the interaction term (*β* = 0.039, *p* = 0.789). In line with our hypotheses, there was a significantly higher object focus in Münster than in Kumbo (see Figure 2C/D).

#### Simple Set

For the simple set, there was no significant regression model, *F*(3, 99) = 1.108, *p* = 0.349, with an adjusted *R*^2^ of 0.003. On a descriptive level, object scores were slightly higher in the Münster sample than in the Kumbo sample (see Figure 2E).

#### Consistency of Attentional Styles Across Set

In order to analyze whether the different sets of stimuli index participants’ attentional style more generally, we calculated the internal consistency between the object scores of the four sets. Cronbach’s α for age-corrected residuals was *α* = 0.604 in Münster and *α* = 0.556 in Kumbo.

## Discussion

The present study aimed at investigating how the familiarity of stimuli affects cross-cultural differences in context sensitivity expected along the social orientation hypothesis. For this purpose, participants from Münster in urban Germany and Kumbo in rural Cameroon saw four different sets of stimuli varying in familiarity while their spontaneous gaze behavior was recorded in an eye-tracking paradigm.

Overall, we found support for the differential familiarity hypothesis and partial support for the social orientation hypothesis: When the stimuli were highly familiar for both cultural contexts, we found a significantly higher object score for participants from Münster than for participants from Kumbo. These results are in line with the social orientation hypothesis that predicts higher context sensitivity in interdependent than in independent cultures (Varnum et al., 2010). While this pattern was significant for the culture-specific matched set, there was a descriptive though non-significant trend for the simple set, with a tendency to higher object scores in Münster than in Kumbo. If, however, the stimuli were systematically less familiar to participants of one of the cultures, the pattern of results changed: For the standard and non-semantic sets, we found a higher object score in Kumbo than in Münster.

This finding – which is unexpected based on the social orientation hypothesis alone – is similar to the findings from Köster et al. (2018), based on samples with a broader age range. Taken together, these findings indicate that the cross-cultural differences in context sensitivity are highly sensitive to the stimulus material used. Specifically, if the overall scene is less familiar, more attention is directed to the focal object, which can lead to reversed findings than would be expected based on the cultural orientation of the participants. When looking at visual stimuli of the type used in this study, namely the standard and the non-semantic stimuli, previous studies have shown that the focal object is fixated first (see Chua et al., 2005; Köster and Kärtner, 2018). If the stimulus is unfamiliar, the exploration of the object may take longer and, thus, due to the limited presentation time, less time remains to explore the context. This might explain why participants look at the object for longer if the stimuli are unfamiliar, as we found for the standard and the non-semantic set in Kumbo.

However, this does not explain the findings for the simple set: although the stimuli are familiar in both cultural contexts – according to the expert judgments, somewhat more so for the Kumbo participants – context sensitivity did not differ between cultures. Two explanations are plausible: first, it might be that – due to the fact that the pictures were taken in Kumbo – the depicted backgrounds, but not the objects, were more typical for Kumbo participants, which led the Münster participants to explore the backgrounds more. As a consequence, the two effects (familiarity and social orientation) neutralized each other, leading to similar results on both cultures. Second, as the background is quite simple in this set (e.g., a tabletop) and does not provide much potential for exploration, the saliency of the object compared to the background might have been even higher. Possibly, culture-specific patterns of context sensitivity may only come into effect if stimuli, including the context, are sufficiently complex. Based on these data, the potential explanations cannot be further scrutinized, but both suggest that the cross-cultural differences in context sensitivity are highly susceptible to the stimulus material used.

Furthermore, we expected age-related changes in attentional styles, namely that context sensitivity would increase with age (Imada et al., 2013). While there was no significant age effect for the simple set and only descriptive trends for the non-semantic and the culture-specific matched set, there was a culture-specific pattern for the standard set. More specifically, context sensitivity increased for the Münster sample but remained at the same level across ages for the Kumbo sample. It is conceivable that the unequal familiarity of the standard set might have influenced the results in the sense that we found the expected age change in Münster, where participants were relatively familiar with the stimuli, while in Kumbo, the unfamiliarity of the presented stimuli might have led to an increased interest in the object across all age groups. Consistent with other studies, the descriptive results consistently hint at an increase in context sensitivity with age. While the *a priori* power analysis (*f*^2^ = 0.15, *α* = 0.05, *β* = 0.80) indicated a minimum sample size of *N* = 77 to detect medium effects of age in the regression analysis, the potential effects seem to be rather small. While our focus was on testing for linear age effects across a broader age range, larger sample sizes are advisable, especially, when interested in contrasting specific age groups around age of emergence.

Finally, we explored whether perceptual styles are consistent across the different stimulus sets. The internal consistencies of the gaze behavior across sets show that all four sets capture similar aspects of participants’ attention. While there was no correlation between the context sensitivity measured with different tasks (i.e., eye-tracking, picture description, and optical illusion) in the study by Köster et al. (2018), the results of this study indicate that the four sets capture a sufficiently similar concept.

It should be noted that it would have been of additional interest to run a fully-balanced design with both groups of participants observing the stimuli of their own and the other culture (i.e., both culture-specific sets). By doing so, it would have been possible to compare the participants’ gaze behavior when looking at the respective unfamiliar set to further back up our results. However, here our main focus was to compare the culture-specific matched set, that is highly familiar in a given culture, to a standard set of stimuli used in other studies analyzing the development of cross-cultural differences in attentional styles, which allows important conclusions about the validity of earlier findings and the influence that stimulus familiarity might have on spontaneous visual attention. A further limitation concerns the unequal AOI sizes of the different sets: When inspecting the absolute object scores across sets, it is important to keep in mind that these cannot be compared directly, but the different AOI sizes between sets may have influenced the respective absolute object scores (e.g., object scores were smallest in the non-semantic set, which also had the smallest AOIs). Having said this, the rationale of this study remains unaffected since the question whether the familiarity of the stimuli affects gaze behavior is addressed by analyzing whether the cross-cultural differences vary by stimulus familiarity.

Taken together, these findings provide considerable potential for further research as they highlight the importance of the stimulus materials and tasks used to assess the concept of context sensitivity across different cultural contexts. For future research, it would be advisable to carefully develop appropriate stimuli reflecting the actual lifeworld of participants from different cultural contexts. In this respect, it should be taken into account that the overall scene of a stimulus, including object and background, is equally familiar across the cultures being compared. In most of the previous studies on analytic and holistic visual attention (e.g., Chua et al. 2005), this issue has not been addressed in detail. In their study, Masuda and Nisbett (2001) expressed the concern that the higher familiarity of the underwater stimuli in Japan compared to the US might have affected the results. However, when adapting stimuli categories in a second study that used wildlife stimuli instead (considered to be more familiar in the US), they replicated their results in a recognition task and did not find any influence of stimulus familiarity. One reason might be that these studies are mainly conducted in urban, industrialized contexts in the Western and East-Asian hemisphere, where differences might be less obvious; this issue might become more apparent when comparing rural and urban contexts (see Caparos et al., 2012). However, to extend findings on cultural differences in analytic and holistic perceptional styles and the underlying mechanisms, it is particularly important to investigate more diverse cultural contexts, and, in this respect, to carefully develop the stimuli used in order to conduct studies in a culturally sensitive and fair way.

Furthermore, in this study we only considered the effect of stimulus familiarity on spontaneous gaze behavior. It remains an open question how stimulus familiarity affects other tasks capturing attentional styles. For example, Senzaki et al. (2014) demonstrated that cultural differences in context sensitivity become especially pronounced when participants are asked to verbally describe visual scenes. Thus, it would be interesting to investigate how the presentation of differently familiar pictures is reflected in the verbal descriptions of participants from different cultural contexts.

This study has shown that stimulus familiarity is a central aspect to take into account when investigating cross-cultural differences in context sensitivity. Specifically, stimulus familiarity should be taken into account when analyzing other aspects that influence perceptual patterns in order to understand the mechanisms behind cross-cultural differences, such as cultural socialization practices. For example, Köster and Kärtner (2018) proposed that perceptual styles are socialized via a verbal route in parent-child interactions during the preschool years. This supports the widely shared assumption that attentional styles are socially transmitted (Masuda and Nisbett, 2001; Senzaki et al., 2016; Masuda, 2017), but specific cross-cultural evidence for this proposal is still missing. In this context, the present results have important implications for the compilation of stimuli in future studies, which are set out to test cross-cultural differences in visual attention.

## Data Availability Statement

The raw data supporting the conclusions of this article will be made available by the authors, without undue reservation.

## Ethics Statement

Ethical approval was not provided for this study on human participants because all details of the ethical guidelines have been followed. This research was conducted in accordance with the Declaration of Helsinki and the Ethical Principles of the German Psychological Society (DGPs), the Association of German Professional Psychologists (BDP), and the American Psychological Association (APA). It involved no invasive or otherwise ethically problematic techniques and no deception and therefore, according to National jurisdiction, does not require a separate vote by a local Institutional Review Board; see the regulations on freedom of research in the German Constitution [§ 5 (3)], and the German University Law (§ 22). Written informed consent to participate in this study was provided by the participants’ legal guardian/next of kin.

## Author Contributions

JK and MK designed the study and the stimuli. MK and RY recruited the sample and conducted the study. SJ analyzed the data. SJ, JK, and MK wrote the manuscript. JK supervised the research. All authors contributed to the article and approved the submitted version.

### Conflict of Interest

The authors declare that the research was conducted in the absence of any commercial or financial relationships that could be construed as a potential conflict of interest.

## Footnotes

^1^https://pixabay.com
